# Polysaccharide from aerial part of *Chuanminshen violaceum* alleviates oxidative stress and inflammatory response in aging mice through modulating intestinal microbiota

**DOI:** 10.3389/fimmu.2023.1159291

**Published:** 2023-04-21

**Authors:** Yuan-Feng Zou, Xiao-Ping JiZe, Cen-Yu Li, Chao-Wen Zhang, Yu-Ping Fu, Zhong-Qiong Yin, Yang-Ping Li, Xu Song, Li-Xia Li, Xing-Hong Zhao, Bin Feng, Chao Huang, Gang Ye, Hua-Qiao Tang, Ning-Yuan Li, Ji Chen, Xing-Fu Chen, Meng-Liang Tian

**Affiliations:** ^1^ Natural Medicine Research Center, College of Veterinary Medicine, Sichuan Agricultural University, Chengdu, China; ^2^ Key Laboratory of Animal Disease and Human Health of Sichuan Province, College of Veterinary Medicine, Sichuan Agricultural University, Chengdu, China; ^3^ State Key Laboratory of Crop Gene Exploration and Utilization in Southwest China, Chengdu, China College of Agronomy, Sichuan Agricultural University, Chengdu, China; ^4^ Animal Nutrition Institute, Sichuan Agricultural University, Chengdu, China; ^5^ College of Agronomy, Sichuan Agricultural University, Chengdu, China

**Keywords:** Chuanminshen violaceum, polysaccharide, antioxidation, antiinflammatory, gut-liver axis, gut microbiota

## Abstract

Aging is a biological process of progressive deterioration of physiological functions, which poses a serious threat to individual health and a heavy burden on public health systems. As population aging continues, research into anti-aging drugs that prolong life and improve health is of particular importance. In this study, the polysaccharide from stems and leaves of *Chuanminshen violaceum* was obtained with water extraction and alcohol precipitation, and then separated and purified with DEAE anion exchange chromatography and gel filtration to obtain CVP-AP-I. We gavaged natural aging mice with CVP-AP-I and performed serum biochemical analysis, histological staining, quantitative real-time PCR (qRT-PCR) and ELISA kit assays to analyze inflammation and oxidative stress-related gene and protein expression in tissues, and 16SrRNA to analyze intestinal flora. We found that CVP-AP-I significantly improved oxidative stress and inflammatory responses of the intestine and liver, restored the intestinal immune barrier, and balanced the dysbiosis of intestinal flora. In addition, we revealed the potential mechanism behind CVP-AP-I to improve intestinal and liver function by regulating intestinal flora balance and repairing the intestinal immune barrier to regulate the intestinal-liver axis. Our results indicated that *C. violaceum* polysaccharides possessed favorable antioxidant, anti-inflammatory and potentially anti-aging effects *in vivo*.

## 1 Introduction

Aging is a process of deterioration of the body’s tissues and organs over time, with a decline in the function of all systems, eventually leading to cellular and even individual death. With age-related diseases such as Alzheimer’s disease, Parkinson’s disease, cardiovascular disease as well as cancer increasing exponentially worldwide in recent years (1–3), there is a constant desire to slow or even reverse aging. Aging is a multifaceted biological process resulted from the combined effect of many factors, and the most widely accepted theory of aging is the free radical theory of aging (4). The free radical theory suggests that increased oxidative stress is an important driver of aging. Under normal physiological conditions, redox levels remain in balance. As we age, the body produces more free radicals (mainly ROS), but the reduced activity of enzymes involved in scavenging free radicals leads to oxidative stress damage (5, 6) and chronic inflammation (7, 8), thus exhibiting the characteristics of aging. One of the main features of aging is the decline in intestinal digestive functions, manifested by disturbances in the intestinal flora, atrophy of the intestinal mucosa and destruction of intestinal immune function (9, 10). This intestinal dysfunction allows microorganisms (or derived metabolites) and toxins (LPS) to enter the circulation and reach the liver, causing or worsening a range of liver diseases (11, 12). Similarly, aging-induced decreases in the metabolic and detoxification functions of the liver affect the intestinal tract through bile acids (13, 14). This bidirectional relationship between the gut and liver *via* the portal vein, known as the gut-liver axis, was first proposed in 1988 and has attracted widespread attention (15). Accumulated studies show that liver and intestinal diseases can be treated through the gut-liver axis theory (16, 17), which shows that the gut-liver axis balance is so important for a healthy body. The gut microbes, which are part of this two-way communication between the gut and liver, are essential for maintaining the homeostasis of the gut-liver axis (18, 19). In recent years, there has been an increasing amount of research into the use of microbiota-targeted dietary and probiotic interventions and novel therapeutic applications such as faecal microbiota transplantation in the prevention and treatment of ageing-related diseases (20). Therefore, screening and identifying potential therapeutic molecules that balance the gut microflora against aging is of great significance.

*Chuanminshen violaceum* Sheh et Shan (CVSS), mainly distributed in Sichuan and Hubei provinces, traditionally used as a medicine from the roots, it is both a bulk Chinese herb and a premium nutritional health product, mostly used as a fitness tonic and as an edible ingredient in folklore for hundreds of years (21). Natural polysaccharides have attracted great interest due to their wide range of sources, low toxicity, few side effects and diverse biological activities (22). Pharmacological studies have shown that polysaccharides are the main active ingredients of CVSS and have various activities such as antioxidant (23), immunomodulatory (24) and antiviral (25). In the industrial production of traditional Chinese medicine, non-medicinal parts, such as stems and leaves of traditional root-based medicines, are generally discarded, resulting in a waste of resources and environmental pollution. These plant parts may be suitable plant resources for the production of phytochemicals (26). Numerous studies have shown that non-medicinal parts of traditional Chinese herbs have the same biological activity as their medicinal counterparts (27, 28). Therefore, in this study, we aimed to evaluate whether CVSS polysaccharide can alleviate oxidative stress injury and inflammatory response in the gut and liver of naturally aging mice, and to investigate the potential function of CVSS polysaccharide in the gut-liver axis and their correlation with gut microbiota to provide evidence for the further development and utilization of CVSS.

## 2 Materials and methods

### 2.1 Materials

The stems and leaves of *Chuanminshen violaceum* were collected from Bazhong City, Sichuan Province, in April 2022 and identified by Dr Yuan-Feng Zou, College of Veterinary Medicine, Sichuan Agricultural University. The fresh stem leaves are treated in an oven at 105°C for 15 minutes to destroy and blunt the oxidase activity in the leaves by high temperature, inhibit the enzymatic oxidation of tea polyphenols etc. in the fresh leaves and evaporate some of the water in the fresh leaves, then dried at 55°C to constant weight and sheared into flakes then dried at 55°C to constant weight and sheared into pieces

### 2.2 Isolation, chemical composition, monosaccharide composition and average-molecular weight of CVSS polysaccharides

The extraction of polysaccharides was carried out based on the previous method (29). Briefly, the dried stems and leaves of *Chuanminshen violaceum* (100g) were pre-treated by 90% ethanol (v/v) to remove impurities until colorless, then the dried residues were treated with boiling water under the following conditions: 100°C, 2 h, solid–liquid ratio 1:20, 2 times. The extract was extracted by filtration, alcohol precipitation, dialysis (cut off 3500 Da) and loaded to a lyophilizer (LGJ-10G, Beijing Sihuan Qihang technology Co., Ltd., Beijing, China) to obtain CVSS crude polysaccharide (CVP) powder. For further purification, CVP (400 mg) was dissolved in distilled water (20 mL), centrifuged, filtrated (0.45 µm) and applied into an anion-exchange column packed with DEAE- Sepharose Fast Flow (50 mm × 40 cm; Beijing Rui Da Heng Hui Science Technology Development Co., Ltd, Beijing, China). The neutral and acidic fractions were eluted with 2 L distilled water followed by 0–1.5 mol/L NaCl solution at a flow rate of 2 mL/min, respectively. The elution profiles of acidic fractions were monitored by the phenol–sulfuric acid method (30), and the acid polysaccharide fraction was collected according to the elution profiles, followed by dialysis (cut off 3500 Da) to remove NaCl, concentrating and freeze-drying, named as CVP-AP. Finally, CVP-AP was further purified by gel filtration (Sepharose 6FF, 2.5 cm × 100 cm, Beijing Rui Da Heng Hui Science Technology Development Co. Ltd) to obtain a purified fraction (31), named CVP-AP-1, because it was more purified than CVP-AP, CVP-AP-I was collected and lyophilized for subsequent studies.

The total carbohydrate content of CVP-AP-I was determined by the phenol sulfuric acid method (30), the total amounts of phenolic compounds and proteins of CVP-AP-I were determined by Folin-Ciocalteu (32) and Bio-Rad protein assay (33). Referring to previous research methods (34), the monosaccharide composition of CVP-AP-I was determined by HPLC (Agilent Technologies, 1260 Infinity II, USA). Briefly, CVP-AP-I was dissolved in TFA (trifluoroacetic acid) and then hydrolyzed at 100°C for 8 h. After repeated evaporation and concentration with methanol to remove excess TFA, the sample was then derivatized with NaOH and PMP (1-phenyl-3-methyl-5-pyrazolone) -methanol at 70°C for 100 min, and finally the pH was adjusted to neutral and extracted with chloroform to obtain the sample derivative. the standard monosaccharide mix including mannose (Man), ribose (Rib), Rhamnose (Rha), glucuronic acid (GlcA), galacturonic acid (GalA), glucose (Glc), galacturonic (Gal), xylose (Xyl) and arabinose (Ara) were derivatized at the same manner. All derivatives were chromatographed on HPLC equipped with C18 column (250 nm ×4.6 mm, 5 μm), with following settings: mobile phase: 0.05 mol/L phosphate buffer (NaH2PO4–Na2HPO4, pH6.8)-acetonitrile (82:18, V/V); flow rate: 1.0 mL/min; column temperature 30°C; detection wavelength of DAD was 250 nm; injection volume: 20 μL.

The molecular weight (Mw) of CVP-AP-I was determined by gel permeation chromatography method (Waters 515 liquid chromatography pump, Waters 2410 differential detector (Refractive Index), Waters Ultrahyrdogel Linear gel column (300 × 7.8 mm) (34, 35). The Mw control dextran (2.5–5348 Da) and CVP-AP-I were dissolved in the mobile phase. Sample solutions were injected after the system was stable, and the resulting chromatogram was recorded. The weight-average molecular weight (Mw), number-average molecular weight (Mn) and their distribution equivalents were obtained by the standard curve. The chromatographic conditions are as follows: the mobile phase was 0.2 mol/L NaNO3 solution, pH = 6.0; flow rate was 0.6 mL/min; the column temperature: 40°C; the injection volume was 20 µL.

### 2.3 Animal care and experimental design

**Figure 1:**
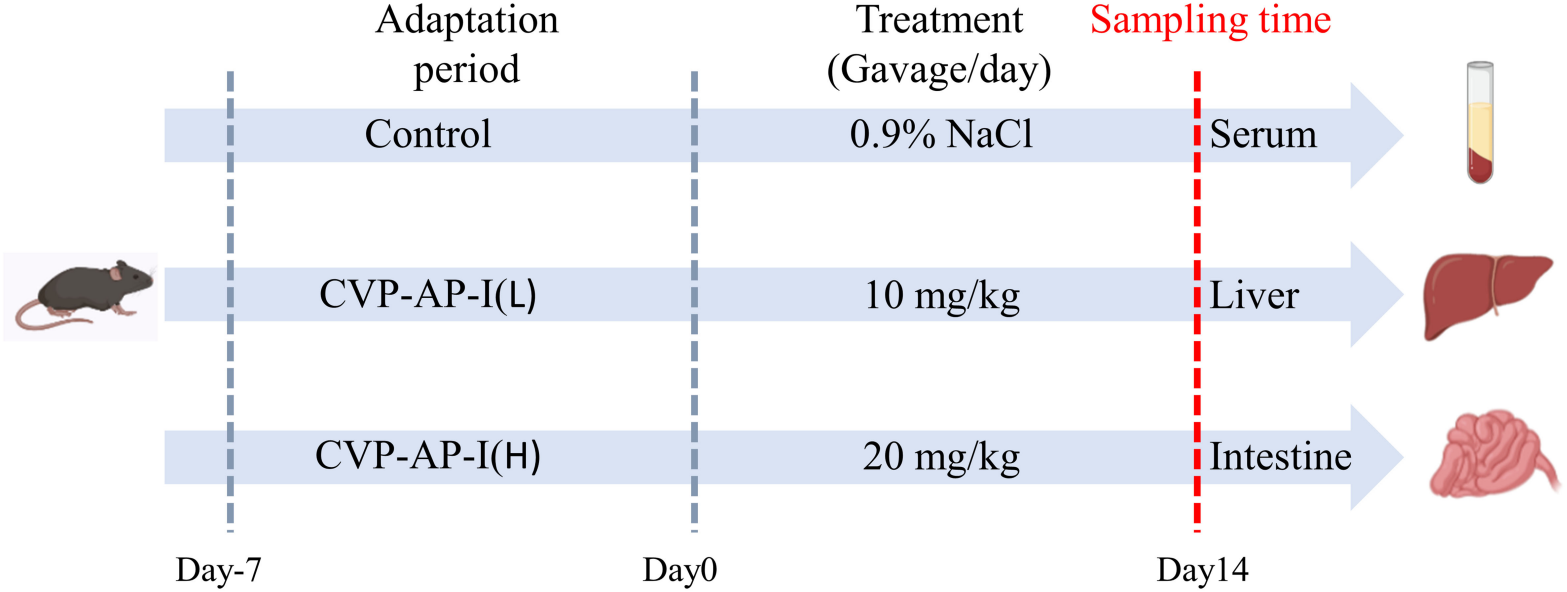
Schematic overview of experimental design.

All animal studies were conducted in accordance with the Animal Care and Use Committee guidelines of Sichuan Agricultural University. Thirty male specific-pathogen-free C57BL/6 mice (48 weeks old) were purchased from Beijing Vital River Laboratory Animal Technology Co., Ltd (Beijing China), and housed in a pathogen free environment and free access to standard chow and water for 7 days, with an automatically controlled 12 h light/dark cycle, the temperature was 25 ± 2°C and the humidity was 60%. After the acclimatization period, as shown in **Figure 1**, all mice were randomly divided into three groups (10 mice in each group), and received a daily gavage of solution (same volume of saline), 10 mg/kg polysaccharide, or 20 mg/kg polysaccharide group for 14 consecutive days. Food intake was recorded daily and body weight was measured every 2 days. At the end of the experiment, all mice were fasted overnight, blood was collected from the eyeballs the next day. Finally, they were euthanized and dissected quickly, then the main organs of liver, intestine tissue, and cecum contents were collected rapidly. All tissue samples were divided into two parts, one was frozen in liquid nitrogen, and then stored at -80 °C for gene expression analysis and biochemical analysis, the other one was fixed in 4% paraformaldehyde for subsequent histopathological examination.

#### 2.3.1 Blood biochemical test

The biochemical indexes of serum, including alanine aminotransferase (ALT), aspartic aminotransferase (AST), triglyceride (TG) and total cholesterol (TC), were detected by automatic hematology analyzer (Shenzhen Icubio Biomedical Technology Co., Ltd.).

#### 2.3.2 Histological staining

The duodenum, jejunum, ileum and liver tissue fixed in 4% paraformaldehyde solution were cut into appropriate size. The tissues were embedded in paraffin and cut into 5um sections, then stained with hematoxylin and eosin according to the manufacturer’s instructions (H&E, G1260, Solarbio, Beijing, China). Brightfield images at 200 × magnification were captured on Nikon Eclipse Ti microscope (Melville, NY, USA).

#### 2.3.3 Quantitative realtime PCR

The intestine (duodenum, jejunum, ileum) and liver tissues were ground under liquid nitrogen. The total RNA was extracted with TRIzol reagent (Biomed, Beijing, China) from the tissue powder, then the RNA (5 μg/μL) was reverse transcribed into cDNA using M-MLV 4 First-Strand cDNA Synthesis Kit (Biomed, RA101-12, China) and further applied to qRT-PCR in a step Oneplus System (Biosystems, Foster City, CA, USA) using determine double-stranded DNA. The procedure conditions were one cycle of 30s at 95°C for pre-denaturation, 40 cycles of 95°C (10 s) and optimum annealing temperature (30s) for denaturation and annealing/extension, respectively. The gene expression levels were calculated according to the regulative relevant quantification method followed by the ΔΔCT method (34). The relative gene expression was normalized to internal control as β-Actin. The primer sequences (Tsingke Biotechnology Co., Ltd, China) for genes expression detection in this study are listed in **Table 1**.

**Table 1:** primer sequences for qRT-PCR.

Gene	Primer sequence 5’ to 3’	PubMed no.	bp
β-actin	F: TCACGGTTGGCCTTAGGGTTC R: CGCTCGTTGCCAATAGTG	NM_001259638.1	71
IL-1β	F: CCTGTGTTTTCCTCCTTGCCT R: AGTGCGGGCTATGACCAATTC	NM_008361.4	158
TNF-α	F:CTCTTCTCATTCCTGCTCGT R: ACCCCGAAGTTCAGTAGACA	NM_012675.3	62
IL-6	F: AAATATGAGACTGGGGATGTC R: TCAGTCCCAAGAAGGCAAC	NM_001314054	90
CAT	F: ACCAGATACTCCAAGGCAAA R: TAAAATTTCACTGCAAACCCC	NM_009804.2	137
SOD1	F: GAACCATCCACTTCGAGCAG R: ATCACACGATCTTCAATGGAC	NM_011434.2	265
GPX	F: TGCTTGCCTCCTAAATGCTG R: CCCAGAATGACCAAGCCAA	NM_001329860.1	81
Nrf2	F: AACCTCCCTGTTGATGACTTC R: CTGTCGTTTTCTCCCTTTTCTC	NM_001399226.1	76
Muc2	F: TCATCAACCTTCACTACCCCA R: TTTTGCACACTAACCCAAC	NM_023566.4	247
ZO-1	F: TCGATCAAATCATTACGACCCT R: GCTCTCAAAACTTCTTCGGTCAA	NM_001352638.1	55
Occludin	F: TTGAAAGTCCACCTCCTTACAGA R: CCGGATAAAAAGAGTACGCTGG	NM_001360536.1	129

#### 2.3.4 Determination of oxidative stress indicators, inflammatory factors, LPS and sIgA

The concentrations of inflammatory factors (IL-6, IL-1β, TNF-α) and LPS in serum, liver and jejunum, and sIgA in jejunum were determined by ELISA kits according to the manufacturer’s instructions (MlBio, Shanghai, China). The levels of SOD (superoxide dismutase), CAT (catalase), GPX (glutathione peroxidase) and MDA (malondialdehyde) in serum, jejunum and liver tissues, as well as ROS (reactive oxygen species) and T-AOC (total antioxidant capacity) levels in tissues were analyzed with biochemical kits according to the manufacturer’s instructions (Nanjing Jiancheng Biotechnology Institute, China). the total protein concentration was assessed according to the BCA assay kit (Beyotime, Shanghai, China).

#### 2.3.5 Gut microbiota analysis

Fresh digesta isolated from the cecum was quickly sent to Novogene Technology Co., Ltd. (Beijing, China), under dry ice conditions for gut microbiota analysis. The DNA was extracted using CTAB/SDS (cetyltrimethylammonium bromide/sodium dodecyl sulfate) Bromide method, and diluted to 1 ng/μL using sterile water. The V4 region of 16S rRNA was amplified by PCR using specific primers (515F: CCTAYGGGRBGCASCAG; 806R: GGACTACNNGGGTATCTAAT), followed by mixing and purification of PCR products, and library construction using TruSeq^®^ DNA PCR-Free Sample Preparation Kit. The libraries were quantified by Qubit and Q-PCR, and then sequenced using NovaSeq 6000.

### 2.4 Statistical analysis

Data represent the mean ± standard deviation (SD) or mean ± standard error of the mean (SEM). One-way ANOVA with LSD *post-hoc* test was performed by using SPSS 27.0 software. The p-value of 0.05 or less was considered with statistically significance.

## 3 Results

### 3.1 Isolation, chemical composition, monosaccharide composition and average-molecular weight of CVSS polysaccharide

**Figure 2:**
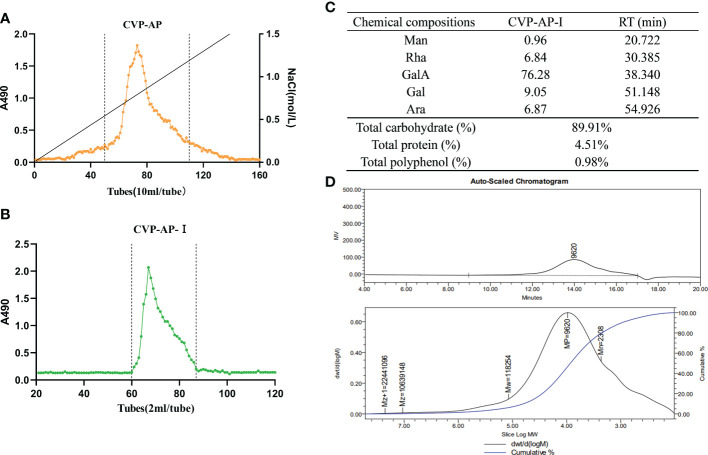
Isolation, chemical composition, monosaccharide composition and average-molecular weight of CVSS polysaccharides. **(A)** The elution curve of CVP-AP on DEAE anion exchange chromatography. A single component is obtained. **(B)** CVP-AP-I elution profile was obtained after gel filtration purification. **(C)** The chemical compositions of CVP-AP-I including monosaccharide composition (mol %), total carbohydrate (%), protein content (%), polyphenol content (%). **(D)** The molecular weight determination of CVSS polysaccharide by gel permeation chromatography.

Crude polysaccharide (CVP) was obtained from the stems and leaves of CVSS by water extraction and alcohol precipitation, with a yield of 5.06%. There are no previous reports related to polysaccharides from the stems and leaves of CVSS, but the extraction rates of crude polysaccharides from CVSS leaves were reported to be in the range of 4.73%-5.41% (36), which were similar to our extraction rates. CVP was separated by DEAE anion exchange chromatography to obtain an acidic component CVP-AP (**Figure 2A**), but the neutral component isolation was less than 1%, so it was not included in the follow-up study. CVP-AP was further purified by gel filtration to obtain CVP-AP-I with a yield of 81.93% (**Figure 2B**). The monosaccharide composition of CVP-AP-I was determined by HPLC and the results (**Figure 2C**) showed that the monosaccharides present in CVP-AP-I were mainly galacturonic acid (GalA), galactose (Gal), arabinose (Ara), rhamnose (Rha), and a small amount of mannose (Man). The total carbohydrate content of the CVP-AP-I was 89.91%, the total protein was 4.51%, and the total polyphenol content was 0.98% (**Figure 2C**). Compared with the reported polysaccharides from the roots and leaves of CVSS, the monosaccharide types and molar ratios of CVP-AP-I were different (36, 37). The weight-average molecular weight (Mw) of CVP-AP-I was 118.2 kDa, as determined by gel permeation chromatography, and a single symmetric peak was observed, as shown in **Figure 2D**.

### 3.2 CVP-AP-I protects naturally aging mice from oxidative stress and inflammation

**Figure 3:**
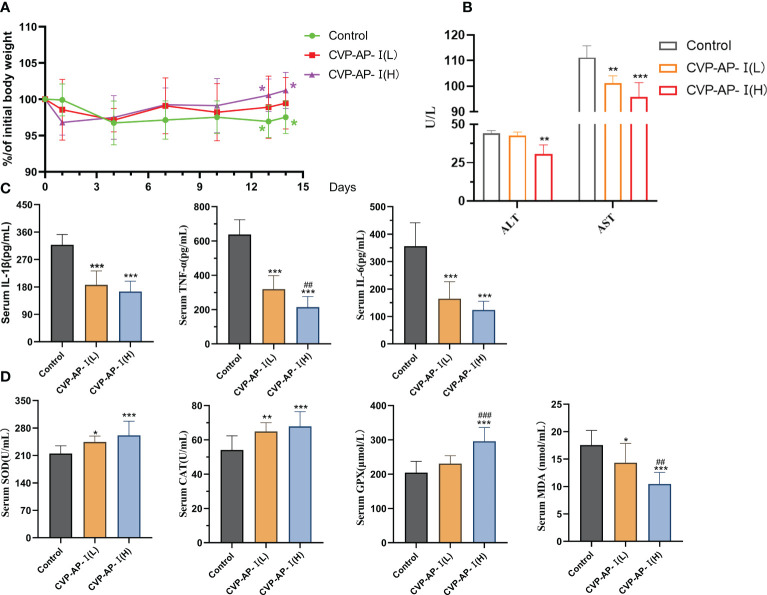
Effects of CVP-AP-I on body weight and serum levels of ALT, AST, pro-inflammatory factors (IL-6, IL-1β, TNF-α), antioxidant enzymes (SOD, CAT, GPX) and MDA in aging mice. **(A)** Changes in body weight of mice (Relative to initial body weight; n=10); **(B)** Quantification shows the serum levels of ALT and AST in the mice of different groups (n=4); **(C)** Quantification shows the inflammatory factor IL-1, TNF-α, IL-6 level in the serum from different groups mice(n=6). **(D)** Quantification shows the activity of the antioxidant enzymes SOD, CAT and GPX and the level of MDA in the serum of the mice in the different groups (n=6). All data are represented as means ± SD.*p<0.05, **p<0.01, and ***p<0.001 as compared with Control Group. ##*p*<0.01 and ###*p*<0.001 as compared with CVP-AP-I (L) group.

As we all know, aging is the inevitable process of life. It is characterized by progressive irreversible degenerative changes in the structure and function of the organs with age, under the influence of multiple factors, accompanied by oxidative stress and inflammatory reactions (7, 8, 38, 39). Therefore, drugs that reduce oxidative damage are the first choice for anti-aging. A mouse’s life cycle is typically 18 ~ 24 months, with aging-related biomarkers slowly appearing in mice from the 10th month onwards. The mice used in the experiments were nearly 12 months of age, when most of the biomarkers had already started to show changes. Therefore, we used naturally aging mice as a model to study the roles of the CVP-AP-I in the natural aging process of the organism. After two weeks of polysaccharide intragastric administration, the body weight of mice in the polysaccharide group was greater than that in the control group (**Figure 3A**). Finally, there was a significant increase in body weight in the 20mg/kg polysaccharide group, and no difference in food intake was observed between the three groups. In addition, in the aging process, the activities of liver function indicators ALT and AST will continue to rise with the decline of liver function (40). We quantified the activities of ALT and AST in serum, and found that the activities of ALT and AST significantly decreased in polysaccharide group, especially in CVP-AP-I(H) group (**Figure 3B**). In order to further illustrate the effect of CVSS polysaccharides on aging mice, the serum indicators of inflammation and oxidative stress were quantitatively analyzed. Compared with the aging group, the levels of inflammatory factors IL-1β, TNF-α and IL-6 (**Figure 3C**) and MDA in serum (**Figure 3D**), significantly decreased in the polysaccharide treatment group, while the activities of antioxidant enzymes SOD, CAT and GPX in serum significantly increased (**Figure 3D**). Previous studies on anti-aging drugs mainly focused on their antioxidant activity, such as astragalus polysaccharide inhibiting D-galactose-induced oxidative stress in mice by upregulating antioxidant factors (41). Inflammation induced by oxidative stress is also another characteristic change of aging. CVSS polysaccharide not only up-regulated serum levels of antioxidant factors but also down-regulated levels of inflammatory factors. All these data indicated that different doses of CVSS polysaccharide interventions in naturally aging mice exhibited great antioxidant activity and anti-inflammatory activity, and may had some ameliorative effects on liver function.

### 3.3 CVP-AP-I attenuates oxidative stress and inflammation in the gut and liver of naturally aging mice

**Figure 4:**
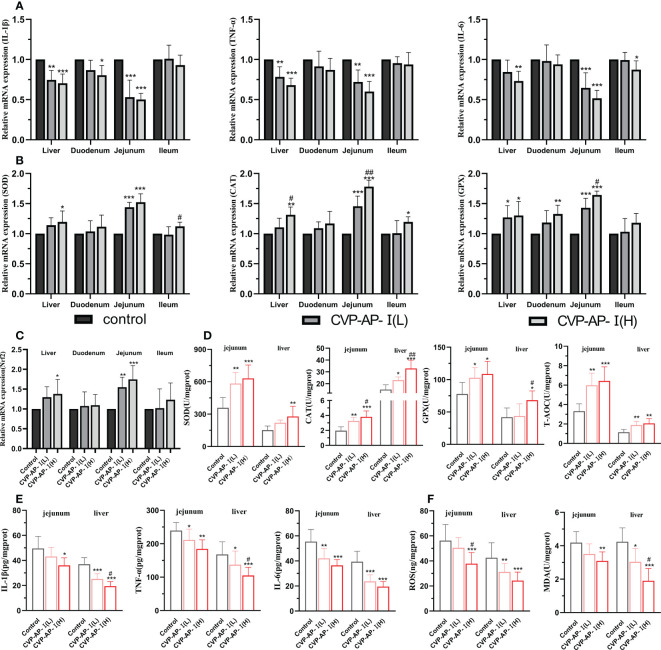
Effects of CVP-AP-I on antioxidant and anti-inflammatory capacity of intestine and liver in naturally aging mice. **(A)** qRT-PCR results show the relative expression levels of inflammatory factors IL-1β, TNF-α and IL-6 genes in liver and intestinal tissues (duodenum, jejunum and ileum) of different groups of mice (n=5); **(B)** qRT-PCR results show the relative expression levels of the antioxidant enzymes SOD, CAT and GPX genes in liver and intestinal tissues (duodenum, jejunum and ileum) of different groups of mice (n=5); **(C)** qRT-PCR results show the relative expression levels of transcription factor Nrf2 gene in liver and intestinal tissues (duodenum, jejunum and ileum) of different groups of mice (n=5); **(D)** Quantitative results show the protein expression levels of antioxidant enzymes SOD, CAT, GPX and T-AOC in jejunum and liver of mice in different groups (n=6); **(E)** Quantitative results show the protein expression levels of inflammatory factors IL-1β, TNF and IL-6 in jejunum and liver of mice in different groups (n=6); **(F)** Quantitative results show the protein expression of ROS and MDA in jejunum and liver of mice in each group (n=6); All data are represented as means ± SD. *p<0.05, **p<0.01, and ***p<0.001 as compared with Control Group. #*p*<0.05 and ##*p*<0.01 as compared with CVP-AP-I (L) group.

Systemic chronic inflammatory responses and oxidative stress are the main features of aging, with a decline in intestinal digestive function, which in turn affects the function of other organ tissues (42). Although, CVP-AP-I could significantly reduce the levels of serum inflammatory factors and liver function indicators ALT and AST, and significantly increase the activity of serum antioxidant enzymes. However, polysaccharides as macromolecules may mainly relay on its indirect effect to liver and the intestine, as reported by our group and other researchers (43, 44). Therefore, we further evaluated the effect of CVP-AP-I on antioxidant and anti-inflammatory capacity of gut and liver in naturally aging mice. We observed a decrease trend in the expression of pro-inflammatory genes (IL-1β, TNF-α and IL-6) and an increase trend in the gene expression of antioxidant enzyme (SOD, CAT and GPX) in the liver, duodenum, jejunum and ileum of aging mice in the polysaccharide-treated groups (**Figures 4A, B**). Nrf2, an important transcription factor that widely exists in various organs of the body, is considered to be a key transcription factor in regulating cells against xenobiotics and oxidative damage (45, 46); and on the other hand, scholars have found that Nrf2 also plays a key role in inflammatory repair (47, 48). Polysaccharides have been shown to reduce oxidative stress and inflammatory responses by activating Nrf2 (49, 50). We found that the relative expression of the Nrf2 gene was significantly higher in the jejunum and liver of the CVSS polysaccharide group (**Figure 4C**), and that the increased expression of antioxidant enzymes and reduced pro-inflammatory factors could be associated with the increase in Nrf2. Although there was a tendency for the polysaccharide-treated groups to alleviate aging-induced oxidative stress and inflammation in the duodenum and ileum, the effect was not significant, so we speculated that the site of utilization of CVSS polysaccharides in the small intestine was primarily in the jejunum. To further illustrate the anti-inflammatory and antioxidant effects of CVP-AP-I, we then quantified the protein levels of inflammatory factors and antioxidant enzymes in jejunum and liver. As shown in (**Figures 4D, E**), at the protein level, compared with the control group, CVP-AP-I significantly increased the activities of antioxidant enzymes (SOD, CAT, GPX) and T-AOC in jejunum and liver, and significantly decreased proinflammatory factors (IL-1 β, TNF- α, IL-6) levels, which was consistent with the qRT-PCR results. Finally, we quantified ROS and MDA protein levels in the jejunum and liver. Specific increases of ROS level have been demonstrated as potentially critical for induction and maintenance of cell senescence process (51, 52). And the MDA content is an important parameter reflecting the body’s potential antioxidant capacity, which can amplify the effect of reactive oxygen species and can reflect the rate and intensity of lipid peroxidation in the body, as well as indirectly reflecting the degree of tissue peroxidative damage (53). Additionally, we found that ROS and MDA were significantly lower in the jejunum and liver of the polysaccharide-treated group compared to the control group, and showed a polysaccharide concentration dependence (**Figure 4F**). These data collectively show the benefits of the CVSS polysaccharide in improving oxidative stress and inflammatory responses in the gut-liver axis of aging mice.

### 3.4 CVP-AP-I ameliorates liver and intestinal tissue defects and repairs the intestinal immune barrier of naturally aging mice

**Figure 5:**
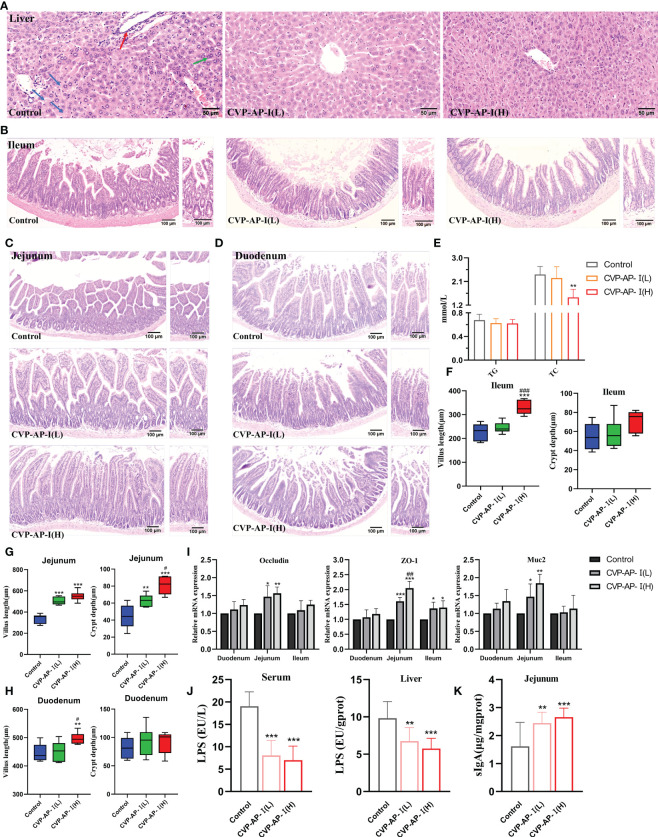
Effects of CVP-AP-I on liver and intestinal histology and intestinal immune barrier in naturally aging mice. **(A)** Representative images of H.E staining show that CVP-AP-I improves age-induced liver lesions. (“**→**” Inflammatory cell necrosis of the liver; “**→**” Multinucleated hepatocytes; “**→**” Mild steatosis of hepatocytes); **(B-D)** Representative images of H.E staining show that CVP-AP-I reverses aging-induced villus and crypt defects in the ileum, jejunum and duodenum; **(E)** Quantification shows the levels of TG and TC in the serum of different groups of mice(n=4); **(F-H)** Quantitative show of CVP reversal of aging-induced intestinal villus and crypt defects in the ileum, jejunum and duodenum. Error bars indicate Mix to Max (n = 6); **(I)** qRT-PCR results show the expressions of Occludin, ZO-1 and Muc2 in duodenum, jejunum and ileum of mice from different groups (n=4); **(J)** Quantification shows LPS levels in the serum and liver of different groups of mice (n=4); **(K)** Quantification shows sIgA levels in the jejunum of different groups of mice (n=6). All data are expressed as mean ± SD. *p<0.05, **p<0.01, and ***p<0.001 as compared with Control Group. #*p*<0.05, ##*p*<0.01, and ###*p*<0.001 as compared with CVP-AP-I(L) group.

Our experimental results show that CVP-AP-I not only alleviates intestinal inflammation and oxidative stress, but also has a similar effect on the liver. While the target organ of polysaccharides is the intestine, the intestinal microbiota, intestinal immune response and intestinal permeability are also related to the function of polysaccharides (54). The intestinal tract has been considered an important target organ mediating the body’s lifespan extension due to its immune and nutritional intake functions. The decline of digestive and absorption functions in the elderly is caused by histological degeneration of the gastrointestinal tract (55), so we speculate that the protective effects of CVP-AP-I on the gut and liver are mediated through the gut-liver axis. To confirm this speculation at the histopathological level, we first performed H.E staining of the liver and intestine. Subsequently, we found that the livers of aging control mice were structurally disorganized and showed aging features, with inflammatory necrosis of hepatocytes, multinucleated cells and mild steatosis. In contrast, the hepatocytes of the two groups supplemented with CVSS polysaccharide, especially the high-dose group, were neatly arranged in a cord-like arrangement with clear hepatic sinusoidal structures (**Figure 5A**). In many previous studies, triglyceride (TG) and cholesterol (TC) metabolism disorders and accumulation have been reported to be closely related to aging (56, 57). For example, increased ROS during aging has been reported to play an important role in the accumulation of cholesterol in the liver by increasing cholesterol and glucose uptake to increase cholesterol synthesis (58). Given the previous improvement in hepatocyte steatosis by CVP-AP-I, we subsequently quantified TG and TC in serum, and found that the CVP-AP-I(H) group similarly reduced serum levels of TG and TC (**Figure 5E**), which was consistent with the results of hepatic H.E staining. In the intestinal tissues, we noted significant histopathological structural defects in the duodenum, jejunum and ileum of the control mice, including fewer and wide villus and reduced number and depth of crypt. Unsurprisingly, we found that the histopathological structure of the intestine (duodenum, jejunum and ileum) of mice supplemented with polysaccharides was more normal, especially in the jejunum of the CVP-AP-I(H) group (**Figures 5B–D, F–H**).

The small intestinal epithelium is composed of a single layer of cells, associated with each other by tight junctions, and a mucus layer that protects the intestinal epithelial cells surface (59). In physiological situations, it carries out the exchange and transfer of nutrients and acts as a physical barrier, together with intestinal microorganisms and the host immune system, to maintain intestinal homeostasis (60). Therefore, tight junctions mediated by the integral membrane proteins, peripheral membrane proteins and mucoprotein are essential for the function of the intestinal epithelial barrier, and their defects lead to impaired intestinal permeability and possible translocation of toxic compounds or pathogenic bacteria within the lumen, causing further local or systemic inflammation and many human diseases (61, 62). Intestinal barrier function decreases with age (63, 64), with qRT-PCR results we found that the gene expression of integral membrane protein (Occludin), peripheral membrane protein (ZO-1), and mucoprotein (Muc2) were all elevated in the small intestine of mice in the polysaccharide consumption group (**Figure 5I**), indicating that CVP-AP-I had an ameliorative effect on intestinal permeability in aging mice. Consistent with this, we detected that CVP-AP-I decreased LPS content in serum and liver (**Figure 5J**), and promoted the production of sIgA (**Figure 5K**), a basic and essential enteroendocrine immune antibody. These results suggest that CVSS polysaccharides can improve intestinal and hepatic structural defects and promote the development of the intestinal mucosal immune system.

### 3.5 CVP-AP-I regulates gut–liver axis through modulating gut microbiota

**Figure 6:**
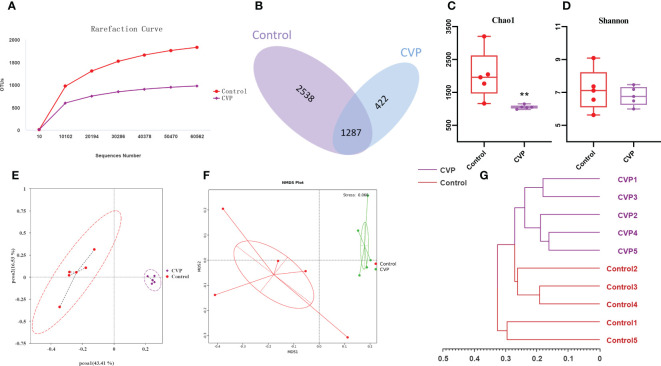
CVSS polysaccharides modulate the composition and structure of gut microflora. **(A)** OUT Rarefaction Curves of gut microbiota in different groups; **(B)** Venn diagram showing the unique and shared OTUs from different groups; **(C)** Bacterial community richness measured by Chao1 index in different groups; **(D)** Bacterial community diversity measured by Shannon index in different group; **(E)** Unweighted Unifrac Principal Coordinate Analysis by bacterial microbiota; **(F)** NMDS ordination based on Bray-Curtis similarities of bacterial communities; **(G)** UPGMA clustering tree based on unweighted Unifrac distances. “*” stands for the comparation with Control; **p<0.01 by one-way ANOVA.

Intestinal microbial-host interactions are critical in the development and maintenance of body immunity (65). During aging, oxidative stress affects the living environment of intestinal microbes and disrupts the structure and function of intestinal flora, leading to the dysregulation of intestinal flora, and further exacerbates oxidative damage and lipid metabolism disorders (66), thereby aggravating the morbidity and mortality of various chronic diseases. Accumulated studies show that anti-aging can be achieved by regulating the structure and composition of intestinal microbiota (67, 68). As CVSS polysaccharides are not directly available to the organism as macromolecules, we evaluated whether the gut flora was its target and assessed the composition of the gut flora by sequencing the 16S rRNA V4 region of the gut bacteria. The previous data showed that the high-dose polysaccharide administration group had the best anti-aging effect, so the intestinal flora analysis was conducted only for the control group and the high-dose group (named CVP group). As shown in **Figure 6A**, we first calculated the sample Rarefaction Curves, where the gradual flattening of the curves indicated a reasonable amount of sequencing data and indirectly reflected differences in the abundance of species in different groups respectively (69). Venn diagram analysis revealed 3825 and 1709 OTUs each in the control and CVP groups, with their respective unique OTUs numbers being 2538 and 422, indicating differences in the structure of the gut microflora between the two groups (**Figure 6B**). Then, we calculated the Chao1 and Shannon indices (70) to assess the richness and diversity of the gut microbiota, and found that they both decreased in the CVP group and Chao1 significantly decreased (**Figures 6C, D**). To determine the differences in gut microbiota structure between the different groups, Unifrac distance-based Principal Co-ordinate analysis (PCOA) (71), Bray-Curtis distance-based metric-free multidimensional calibration method analysis (NMDS) (72) and UPGMA cluster analysis (73) were performed. We found that the CVP group was completely separated from the control group in both the PCoA and NMDS analyses (**Figures 6E, F**), and the community composition of the samples was more similar. Similarly, in the UPGMA cluster tree analysis, we found that samples from the polysaccharide group tended to cluster together, while samples from the control group were scattered or clustered (**Figure 6G**).

**Figure 7:**
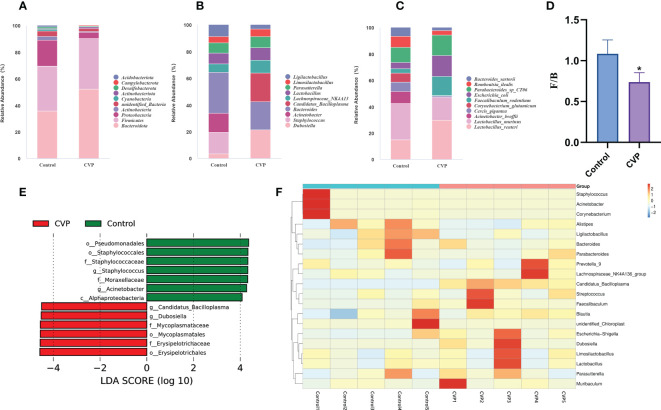
Comparative analysis of the effects of CVSS polysaccharide supplementation on the gut microflora. **(A-C)** Relative abundance of species in the top 10 of the intestinal flora at the phylum, genus and species level; **(D)** Quantification shows the abundance ratio of Firmicutes/Bacteroidetes at the phylum level, n=5, “*” stands for the comparation with Control; *p<0.05 by one-way ANOVA; **(E)** The histogram of the distribution of LDA values shows the species with significant differences in abundance in different groups; **(F)** Heatmap depicting the relative abundance of 20 bacterial species significantly enriched in different samples at the genus level.

Furthermore, we studied the taxonomic distribution of abundant bacteria at three levels: phylum, genus and species. At the phylum level (**Figure 7A**), the control group was mainly composed of *Bacteroidota* (33.80%), *Firmicutes* (32.07%), *Proteobacteria* (18.45%), *unidentified_Bacteria* (3.58%) and *Actinobacteria* (2.86%), while the CVP group consisted of *Bacteroidota* (50.25%), *Firmicutes* (36.64%), *Proteobacteria* (4.69%), *unidentified_Bacteria* (2.11%), and *Actinobacteriota* (1.33%). Studies have shown that phylum horizontal *actinomycetes*, *Proteobacteria* and *Verrucomicrobia* increased significantly and *Firmicutes* decreased during aging (74, 75). However, CVSS polysaccharide could reverse this phenomenon, and the abundance of *Bacteroidetes*(p<0.01) and *Firmicutes*(p>0.05) in our polysaccharide group increased, while the abundance of *Proteobacteria*(p<0.01) and *actinomycetes* (p>0.05) decreased. We then calculated the abundance ratio of *Firmicutes/Bacteroidetes* (**Figure 7D**), which tended to increase with age and has been suggested as a possible marker of senescence (76). The abundance ratio of *Firmicutes/Bacteroidetes* significantly decreased in CVP group (p=0.023). The analysis of the relative abundance of microbes at the genus level is shown in **Figure 7B**. *Bacteroides* (7.63%) was the most enriched genera in Control group, followed by *Staphylococcus* (3.96%), *Acinetobacter* (3.52%), *Lactobacillus* (1.97%), *Parasutterella* (1.90%), *Lactobacillus* (1.97%) and *Lachnospiraceae_NK4A136_group* (1.53%). *Dubosiella* (6.29%) and *Bacteroides* (6.27%) were the most abundant genera in the CVP group, followed by *Candidatus_Bacilloplasma* (5.38%), *Lachnospiraceae_NK4A136_group* (2.84%), *Lactobacillus* (2.75%) and *Parasutterella* (2.42%). At the species level,we observed higher relative abundance of *Lactobacillus_reuteri* and *Faecalibaculum_rodentium* and lower relative abundance of *Acinetobacter_lwoffii*, *Cercis_gigantea*, and *Corynebacterium_glutamicum* in the CVP group compared to the control group (**Figure 7C**). In addition to *Dubosiella* and *Candidatus_Bacilloplasma*, which increased significantly in the CVP group at the genus level, there were also some increases in the abundance of the genera *Lactobacillus* and *Parasutterella*. Of these, *Lactobacillus* was widely used as one of the most common probiotics (77). Our results showed that *Lactobacillus_reuteri* was the most abundant at the species level in the genus *Lactobacillus*, which protected the micro-ecological balance of the gut by producing reuterin, short-chain fatty acids (SCFA), and lactic acid (78). In addition, *Parasutterella* has been shown to be involved in the maintenance of bile acid homeostasis and cholesterol metabolism (79). All these data indicate that CVSS polysaccharide can regulate the composition and structure of intestinal microorganisms.

To identify key phylotypes of the gut microbiota associated with CVSS polysaccharide function, we used linear discriminant analysis (LDA) to assess cecum microbes in the control and CVP groups. **Figure 7E** shows species with significant differences, indicated by LDA scores > 4.0, reflecting the degree of impact of species that differed significantly between the two groups. At the genus level, the CVP group significantly increased the abundance of *Dubosiella* and *Candidatus_Bacilloplasma* and significantly decreased the abundance of *Staphylococcus* and *Acinetobacter*. *Dubosiella* is isolated and cultured from mouse intestinal contents (80) but there are few studies on its efficacy. It has been reported that *Dubosiella* significantly improves the state of dysglycemia and lipid metabolism in mice and prolongs the life span of nematodes. Surprisingly, in the treatment of high-fat diet-induced obesity (81), alcoholic fatty liver (82), and inflammatory bowel disease (83) with polysaccharides, the abundance of *Dubosiella* was also increased, in the same way as our results, and in some cases predominantly. To further analyse the differences in species abundance, a heat map was generated using the top twenty bacteria in abundance at the genus level for species composition analysis. We found that 19 genera changed in the CVP group compared to the control group, with a trend towards enrichment in 11 genera and a decrease in abundance in 8 genera (**Figure 7F**). Among the genera with increased abundance, *Dubosiella* (80, 84), *Candidatus_Bacilloplasma* (85), *Lachnospiraceae_NK4A136_group* (86), *Faecalibaculum* (87), *Limosilactobacillus* (88, 89), *Lactobacillus* (77) and *Parasutterella* (79) were associated with intestinal homeostasis. Some of the genera with reduced abundance were associated with intestinal inflammation and oxidative stress, such as *Staphylococcus* (90, 91), *Acinetobacter* (92, 93), *Corynebacterium* (94, 95). Therefore, CVSS polysaccharide may regulate the homeostasis of the intestinal flora by increasing the abundance of potentially beneficial bacteria such as *Dubosiella* and *Candidatus_Bacilloplasma*, as well as common beneficial bacteria such as *Lactobacillus*, *Lactobacillus_reuteri* and *Parasutterella*, and decreasing the abundance of harmful bacteria such as *Staphylococcus* and *Fusobacterium*, thereby protecting the normal function of the intestine.

**Figure 8:**
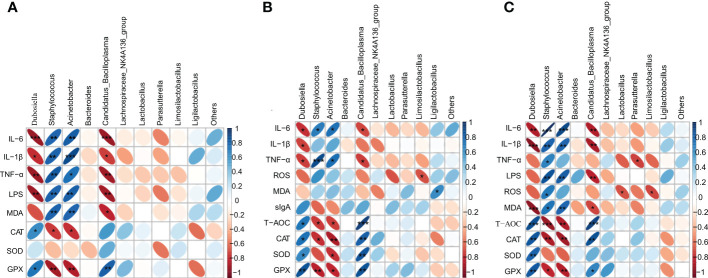
Heatmap showing the correlation of gut microbial genera with pro-inflammatory factors (IL-6, IL-1β, TNF-α), antioxidant enzymes (CAT, SOD, GPX), MDA, LPS, sIgA; **(A)** indicates correlation with serum; **(B)** indicates correlation with jejunum; **(C)** indicates correlation with liver. The colour and shape of the ellipse correlates with the strength of the correlation, with darker and flatter ellipses being more strongly correlated (*p<0.05, **p<0.01, ***p<0.001).

**Figure 9:**
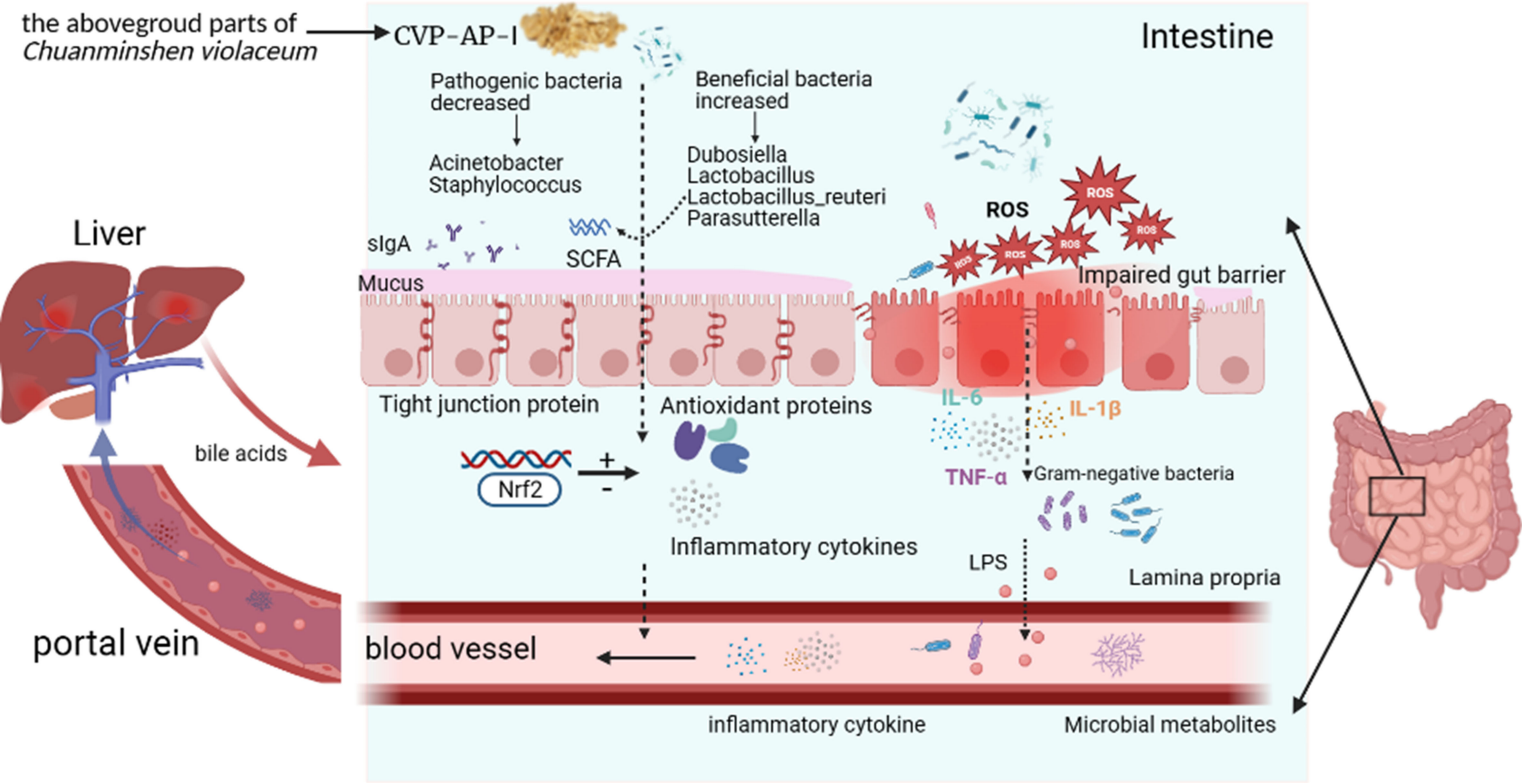
Summary diagram of the pharmacological effects of CVP-AP-I.

Finally, we performed a heatmap correlation analysis using the top ten abundances of intestinal microorganisms at the genus level with pro-inflammatory factors, antioxidant enzymes, MDA, LPS and sIgA in serum (**Figure 8A**), jejunum (**Figure 8B**) and liver (**Figure 8C**). We found that *Dubosiella*, *Candidatus_Bacilloplasma*, *Lachnospiraceae_NK4A136_group*, *Lactobacillus*, *Parasutterella*, and *Limosilactobacillus* were negatively correlated with the levels of pro-inflammatory factors (IL-6, IL-1β, TNF-α), LPS (serum), and MDA in serum, jejunum, and liver, and positively correlated with the levels of antioxidant enzymes (CAT, SOD, GPX) and sIgA (jejunum). *Staphylococcus*, *Acinetobacter*, and *Ligilactobacillus* were positively correlated with the levels of pro-inflammatory factors (IL-6, IL-1β, TNF-α), LPS (serum), and MDA in serum, jejunum, and liver, and negatively correlated with the levels of antioxidant enzymes (CAT, SOD, GPX) and sIgA (jejunum). Seven of the top ten genera in abundance were significantly correlated with inflammatory, oxidative, and other factors, which further verified the above speculated that intestinal flora alleviated oxidative stress and inflammatory response and delayed aging through the gut-liver axis. As shown in **Figure 9**, during the aging process, under the action of ROS, the intestine and liver were damaged by oxidative stress, the intestinal barrier was damaged, the intestinal flora was disturbed, and inflammation was triggered. LPS produced by Gram-negative bacteria and metabolites produced by other microorganisms, and inflammatory factors entered the liver through the intestinal epithelial layer through the portal vein, aggravating the oxidative stress damage in the liver. By promoting the expression of transcription factor Nrf2, CVSS polysaccharide enhanced antioxidant capacity, reduced inflammatory response, repaired intestinal immune damage and regulated dysbiosis, thereby improving oxidative stress damage and inflammatory response in the intestine and liver of naturally aging mice through the intestine-liver axis. The protective effects on the intestine and liver exhibited by the polysaccharide from the stems and leaves of *Chuanminshen violaceum* indicated that the polysaccharides from the stems and leaves had the same good pharmacological activity as the polysaccharides from the medicinal parts of the root, suggesting a predictable application *in vivo* against oxidative stress-related intestinal and liver diseases. In order to further develop and utilize the non-medicinal parts of the ground, more in-depth research is needed.

## 4 Conclusion

In this study, we used DEAE anion-exchange chromatography and gel filtration to obtain a homogeneous acidic polysaccharide, CVP-AP-I, from the stems and leaves of *Chuanminshen violaceum.* The results of chemical composition studies showed that CVP-AP-I is mainly composed of GalA, Gal, Ara, and Rha, and its average molecular weight is 118.2kDa. Subsequently, different doses of CVP-AP-I were orally administered to naturally aging mice, and benefits in terms of oxidative stress and inflammation in the intestine and liver were reported. CVP-AP-I not only improved intestinal function by down-regulating ROS levels and pro-inflammatory factor expression, up-regulating antioxidant enzymes and total antioxidant capacity, repairing structural damage to intestinal tissues and modulating intestinal flora structure, but also has the same effect in liver tissue. These results suggest that CVSS Polysaccharide is an effective modulator and contributes to the homeostasis of the intestinal microbiota and its associated intestinal functions. With improved intestinal flora and intestinal immune barrier, LPS-producing Gram-negative bacteria such as *Acinetobacter* decrease, *Lactobacillus_reuteri*, *Parasutterella*, and possibly the potentially beneficial bacterium *Dubosiella* increase. CVSS polysaccharide reduces serum and hepatic LPS levels, thereby improving oxidative stress and inflammatory responses in the gut and liver through the gut-liver axis.

## Data availability statement

The datasets presented in this study can be found in online repositories. The names of the repository/repositories and accession number(s) can be found below: NCBI Bioproject, PRJNA936077.

## Ethics statement

The animal study was reviewed and approved by the Animal Care and Use Committee guidelines of Sichuan Agricultural University.

## Author contributions

Y-FZ and X-PJ designed the study, C-YL, X-PJ, Y-PF, C-WZ and N-YL finished the experiment; X-PJ drafted the manuscript. L-XL, Z-QY and XS were mainly responsible for the determination of characterization of polysaccharides. X-HZ, H-QT, JC, Y-PL and X-FC detected the bioactivity of polysaccharides. BF and GY performed the graphics and helped to improve the research. Y-FZ, M-LT guided the experiments and reviewed the manuscript. All authors contributed to the article and approved the submitted version.
